# Biotinylated cyclic naphthalene diimide as a searching tool for G4 sites on the genome

**DOI:** 10.1007/s44211-024-00551-5

**Published:** 2024-04-12

**Authors:** Satoshi Fujii, Shinobu Sato, Ryuki Hidaka, Shigeori Takenaka

**Affiliations:** 1 Department of Bioscience and Bioinformatics, Kyushu Institute of Technology, 680-4 Kawazu, Iizuka-Shi, Fukuoka, 820-8502 Japan; 2 Department of Applied Chemistry, Kyushu Institute of Technology, 1-1 Sensui-Cho, Tobata-Ku, Kitakyushu-Shi, Fukuoka, 804-8550 Japan

**Keywords:** G-quadruplex, Cyclic naphthalene diimide (cNDI), Isothermal titration calorimetry (ITC), Pulldown assay, Quantitative polymerase chain reaction (qPCR)

## Abstract

A biotinyl cyclic naphthalene diimide (biotinyl cNDI) (**1**), in which biotin is introduced on the cyclic linker chain of cNDI with high G-quadruplex (G4) specificity, was synthesized. **1** was used for binding analysis to G4 DNAs such as c-myc, c-kit, CEGF, or TA-core. The results showed that **1** bind to G4 DNAs with high affinity and, especially, two molecules of **1** bind to c-myc DNA from top and bottom of G4 site at *K* = 3.9 × 10^−6^ M^−1^ without changing the G4 structure. As a pulldown assay, **1** and streptavidin magnetic beads could be used to recover a c-myc DNA or 120-mer DNA fragment having single c-myc sequence. The qPCR results for the 120-meric DNAs showed that more than 50% of genomic DNA fragments could be recovered by this pulldown assay. The results obtained here might allow the recovery of G4-containing DNA fragments from genomic DNA to analyze the true G4 present in the genome.

**Graphic abstract:**

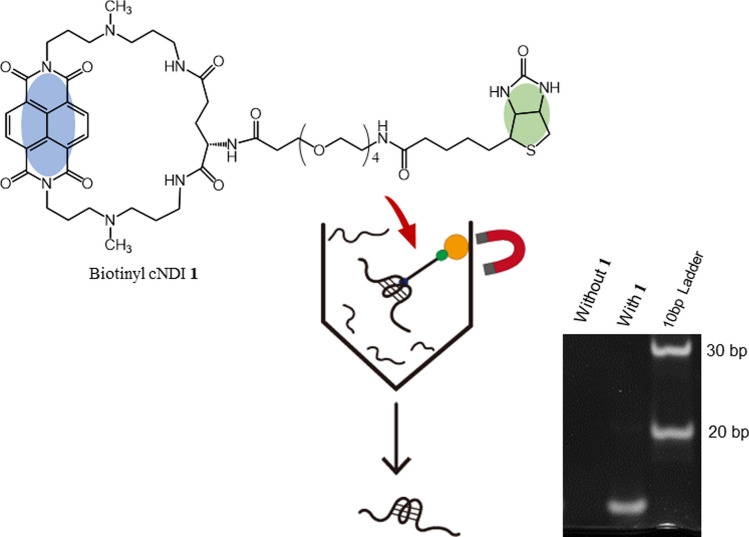

**Supplementary information:**

The online version contains supplementary material available at (10.1007/s44211-024-00551-5).

## Introduction

It has been known that when a large number of guanines are present in single-stranded DNA, G-quadruplexes (G4s) are formed both intra- and inter-molecules. The G4s generated here are known to play important roles in biological processes such as gene transcription and replication [1]. Over 700,000 G4-structures in the human genome have been identified by G4-seq analysis [2]. The analysis revealed the presence of G4 structures in a very large number of gene regions and also showed significant enrichment of G4s in various cancer-related genes. Stabilization of the G4 structure is said to inhibit telomere elongation, suppress oncogene transcription, and destabilize the genome, leading to mutations, deletions, and recombination events [3]. G-quadruplex structures are regarded as emerging therapeutic targets, especially in gene promoter regions. Their stabilization has been linked to the transcriptional repression of oncogenes, making them a novel anticancer strategy [4, 5]. Additionally, G-quadruplexes have been associated with genome instability and DNA damage, which are relevant to cancer therapy [3]. Therefore, G4 ligands that stabilize the G4 structure are considered a promising therapeutic approach for cancer [6]. However, the binding sites of these G4 ligands in the genome have been largely unverified. Elucidating genome-wide G4 ligand-DNA interactions could provide valuable insight into the mechanism of action of this class of agents and facilitate the use of the genome as a therapeutic target.

**Scheme 1:**
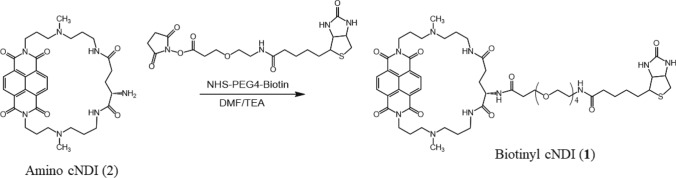
Synthetic route of biotinyl cyclic naphthalene diimide, biotinyl cNDI (**1**)

**Fig. 1:**
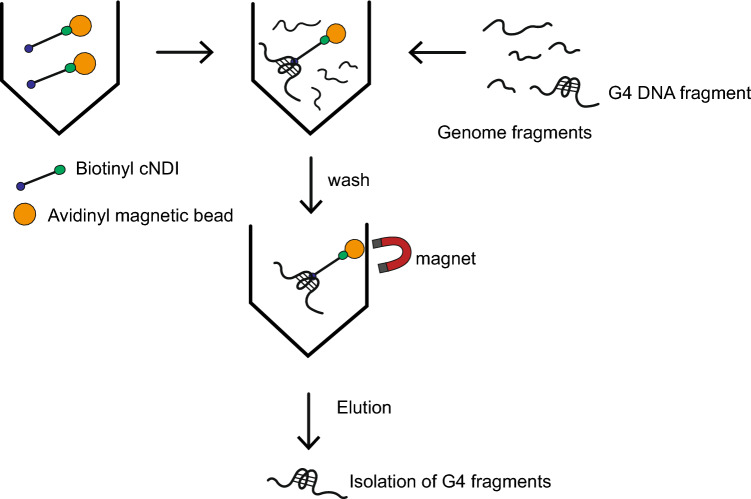
Pulldown protocol using to isolate G4s by biotinyl cNDI, **1**

The genome-wide localization of the active G4 structure was identified by G4-ChIP-Seq, in which DNA fragments containing the G4 formation site were collected by immunoprecipitation and analyzed by sequencing using BG4, an anti-G4 antibody [7]. For G4 ligands, methods attaching biotin to the compound have been established to collect genomic fragments binding to G4 through immunoprecipitation [8–10]. To facilitate the development of pharmaceuticals that specifically target G4, it is important to examine G4 ligands with various binding selectivity. In particular, the use of more selective G4 ligands is expected in this approach. We have achieved a highly selective G4 ligand through our own development of G4 ligands [11] and have attempted to identify G4 structure-forming sites on the genome using this G4 ligand. To explore its potential, here we synthesized a biotinyl cyclic naphthalene diimide (**1**) (Scheme 1) and applied it to a pulldown assay to recover DNA fragments containing G4 (Fig. 1) and its preliminary performance.

## Experimental

### Instruments and materials

The following instruments were used for measurements: JASCO J-820 circular dichroism spectrometer (Jasco, Japan), Hitachi U-3310 spectrophotometer (Hitachi, Japan), or Nano ITC LV (Waters Cop., DE, USA).

Biotin-dPEG4-NHS ester was purchased from Quanta BioDesign (OH, USA), and the synthetic oligonucleotides listed in Table 1 were custom synthesized by Hokkaido System Science (Hokkaido, Japan). 15 µM G4 DNA in 50 mM H_2_KPO_4_-HK_2_PO_4_ buffer (pH 7.0) was kept at 95 °C for 10 min, cooled to 25 °C for 70 min as annealing procedure. The annealed DNA solution was stored at 4 °C. Mixture of 15 µM 12ss(-) and 15 µM 12ss( +) were used as 15 µM dsoligo.

**Table 1:** Oligonucleotides used in these experiments

DNAs	Sequence (5’ to 3’)
c-myc	TGAGGGTGGGTAGGGTGGGTAA
c-kit	AGGGAGGGCGCTGGGAGGAGGG
VEGF	CGGGGCGGGCCTTGGGCGGGGT
TA-core	TAGGGTTAGGGTTAGGGTTAGGG
12-ss (−) 12-ss ( +)	GCGAAACCTCCC GGGAGGTTTCGC
c-myc120	GAGGTATTTCGGGAGGTTTCGCCCCACGCCCTCTGCTTTGGGA-ACCCGGGAGGGGCGCTTATGGTGAGGGTGGGGAGGGTGGGG-AAGGGGGAGGAGAGCGGTATTCGTGGTGTTCGGAGT
c-myc-mut95	GAGGTATTTCGGGAGGTTTCGCCACCTTCTTCACTCTCTTCAC-TCTCTTCATAAGCGCCCCTCCCGGGTTCCCCGGTATTCGTGGT-GTTCGGAGT
F-Primer	GAGGTATTTCGGGAGGTTTCGC
R-Primer	ACTCCGAACACCACGAATACCG

### Synthesis of biotinyl cNDI (*1*)

Amino cNDI (**2**) was synthesized by the previously reported method [9]. 50 mg (84 μmol) of biotin-dPEG4-NHS ester and 173 mg (177 μmol) of **2** were added to 3 mL of super hydrated DMF under a nitrogen atmosphere. Two mL of triethylamine was added and the mixture was stirred at 60 °C under a nitrogen atmosphere for 24 h. After drying under vacuum, the resulting residue was subjected to silica gel column chromatography using the eluent of CHCl_3_:CH_3_OH:diethylamine = 1:0.1:0.1. After collecting the component with *R*_f_ = 0.30, it was removed under reduced pressure and vacuum dried to give 65 mg of yellow material (yield: 70%). Purity of **1** was identified by HRMS, ^1^H-NMR (Fig. S1), and Reverse-phase HPLC (Fig. S2). HRMS (EI^+^) *m*/*z* [M]^+^ Calcd for C_54_H_79_N_10_O_13_S 1107.55, found 1107.55408. ^1^H-NMR (500 MHz, CDCl_3_): *δ* = 1.10 (2H, *t*, 7.5 Hz), 1.81 (12H, m), 1.92–1.96 (10H, m), 2.01 (4H, s), 2.09–2.22 (10H, m), 2.44–2.50 (4H, m), 2.89 (2H, d), 3.31 (1H, m), 3.55 (2H, t), 3.60–3.63 (12H, m), 3.66 (1H, m), 3.74 (1H, m), 4.25 (2H, m), 4.49–4.51 (2H, m), 5.34 (1H, s), 6.07 (1H, s), 6.97 (1H, t), 7.12 (1H, t), 7.35 (1H, d), 7.48 (1H, d), and 8.73–8.78(q, 4H) (Fig. S1).

### Absorption titration of *1* with DNAs

The absorption spectral changes were measured by dropping small amounts of G4 stock solution into 50 mM KH_2_PO_4_-K_2_HPO_4_ buffer (pH 7.0) containing 8 μM **1**. The number of bindings to **1** for G4 and the binding constant were calculated by fitting the theoretical equation of Stootman et al. [13] to Eq. (1), which assumes that n ligands bind in a non-cooperative manner.

1 $$1 - \frac{A}{{A_{0} }} = \frac{1}{2}R_{b} \left\{ {\frac{1}{{K_{a} }} + L_{T} + nS_{T} - \sqrt {\left( {\frac{1}{{K_{a} }} + L_{T} + nS_{T} } \right)^{2} - 4nS_{T} L_{T} } } \right\}$$

where *A*: absorbance, *A*_*0*_: initial absorbance ([DNA] = 0 M), *K*_a_: binding constant, *L*_T_: total ligand concentration *S*_T_: total DNA concentration, n: number of bindings, and *R*_b_: instrument response sensitivity.

### Circular dichroism (CD) measurements

CD spectra were measured in 1.5 µM DNA in 50 mM H_2_KPO_4_-HK_2_PO_4_ buffer (pH 7.0) before and after adding 1.5, 3.0, or 4.5 µM of **1**. The measurement conditions were as follows. Measuring instrument: JASCO J-820 circular dichroism spectrometer, measuring wavelength: 220–500 nm, sensitivity: 100 mdeg, scanning speed: 50 nm/min, response: 4 s, data interval: 0.2 nm, bandwidth: 2 nm, number of integrations: 4, and measurement temperature: 25 °C.

### Tm measurements

Tm were measured in 1.5 µM DNA in 50 mM H_2_KPO_4_-HK_2_PO_4_ buffer (pH 7.0) before and after adding 3.0 µM of **1**. CD absorption changes at 265 nm were measured for G4 DNA, c-myc, c-kit, VEGF, and TA-core under the following conditions: measuring instrument: JASCO J-820, sensitivity: 100 mdeg, response: 1 s, bandwidth, 1 nm, Data acquisition interval: 0.2 °C, integration frequency: 1 time, temperature gradient: 1 °C/min (20–95 °C).

For ds-oligo as double-stranded DNA, the temperature dependence of the change in absorption intensity at 260 nm was measured under the following conditions Data acquisition interval: 0.5 °C, number of integrations: 1 time, temperature gradient: 1 °C /min (25–95 °C).

### Isothermal titration calorimetry (ITC) measurements

The 250 µL of 10 μM DNA in 50 mM H_2_KPO_4_-HK_2_PO_4_ buffer (pH 7.0) was filled into the sample cell with a dedicated syringe, and 50 µL of 50 mM H_2_KPO_4_-HK_2_PO_4_ buffer (pH 7.0) containing 100 μM **1** solution was filled into a dropping syringe. The thermodynamic parameters were calculated by analysis in Independent mode using the data from the 2 to 25th titration, which excluded the values of the first titration. The analysis software was Nano Analyze by TA Instrument. The measurement conditions were as follows—measurement temperature: 25 °C, stirring speed: 350 rpm, cell volume: 180 μL, syringe size: 50 μL, 1–25 titrations: 1.96 μL, and interval: 120 s.

### Pulldown assay for DNAs

The 600 μL of 1 mg/mL beads (Streptavidin MagneSphere® Paramagnetic Particles, Promega) were pretreated according to the instruction manual by using 50 mM H_2_KPO_4_-HK_2_PO_4_ buffer (pH 7.0). The 100 μL of 5 μM **1** and 5 μM DNAs in 50 mM H_2_KPO_4_-HK_2_PO_4_ buffer (pH 7.0) were added to the beads (0.6 mg) and were shaken (125 rpm.) at r.t for 30 min. Then beads were magnetically captured, and the supernatant was collected (sample 1). The beads were washed two times by using 100 μL of 50 mM H_2_KPO_4_-HK_2_PO_4_ buffer (pH 7.0). 100 μL of 1 × PBS was added to the washed beads and shaken at 99 °C for 30 min at 800 rpm. The beads were centrifuged at 11,000 rpm for 5 min at 4 °C, placed on a magnetic stand for 30 s, and 80 μL of supernatant was collected (sample 2).

The 2 μL of sample 1 and sample 2 were measured UV spectra by using NanoDrop ND-1000 (Thermo Fisher Scientific, MA, USA) by using nucleic acid mode.

Native PAGE for sample 1 and sample 2 were performed under the following conditions. Ten μL of sample 1 and sample 2 were mixed with 2 μL of 6 × loading buffer (TakaraBio, Japan), then 5 μL of mixture were casted to 12.5% polyacrylamide gel prepared in 1.25 × TBE (89 mM Tris base, 89 mM borate, and 1 mM ethylenediaminetetraacetic acid, pH 8.0). Gel electrophoresis was run at 200 V for 40 min in 0.7 × TBE. After electrophoresis, the gel was stained with 1 × GelStar® Nucleic Acid Stain (TakaraBio) in 1 × TBE for 30 min and photographed.

### Pulldown assay for c-myc120

The 50 μL of 1 mg/mL beads were pretreated according to the instruction manual by using 50 mM H_2_KPO_4_-HK_2_PO_4_ buffer (pH 7.0). The 5 μL of 1 mg/mL beads, 1 μL of 100 μM **1** and 10 μL of 50 mM H_2_KPO_4_-HK_2_PO_4_ buffer (pH 7.0), and 74 μL Milli-Q water ware shaken (125 rpm) at r.t for 30 min. Then beads were magnetically captured, and the supernatant was discarded. The beads were then washed three times in 50 μL of 50 mM H_2_KPO_4_-HK_2_PO_4_ buffer (pH 7.0) with manual agitation. Beads were resuspended in 90 μL of 50 mM H_2_KPO_4_-HK_2_PO_4_ buffer (pH 7.0) and 10 μL of c-myc120 (10 nM, 1 nM, 100 pM, or 10 pM), c-myc-mut95 (10 nM) and shaken (125 rpm.) at r.t for 30 min, then beads were washed three times same procedure by using the magnet. To release DNA from the beads, 50 μL of MilliQ was added to the washed beads and shaken at 99 °C for 30 min at 800 rpm. The beads were centrifuged at 11,000 rpm for 5 min at 4 °C, placed on a magnetic stand for 30 s, and 40 μL of supernatant was collected.

### qPCR for pulldown DNA

Pulldown DNA (as above, 2 fmol/μL, 200 amol/μL, 20 amol/μL, and 2 amol/μL c-myc120 or 2 fmol/μL c-myc-mut95. Concentration was assuming 100% recovery) was used to quantify via qPCR with a Stratagene Mx3005P (Agilent Technologies) and QuantiTect SYBR Green PCR Kit (QIAGEN). Reaction mixture (25 μL) contained 1 × QuantiTect SYBR Green PCR Master Mix, 0.2 μM F-primer, 0.2 μM R-primer, and 1 μL pulldown DNA. In addition, the standard curve was obtained by performing qPCR of 2 fmol—2 amol c-myc120. Cycling conditions were 95 °C for 25 min, 45 × (95 °C for 15 s, 64 °C for 30 s, 72 °C for 30 s).

## Results

### Interaction of *1* with several DNAs

**Fig. 2:**
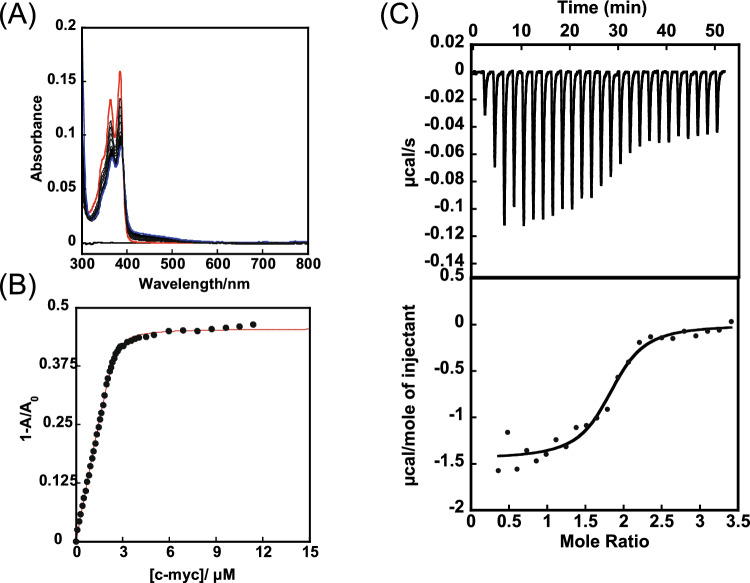
Interaction of biotinyl cNDI with c-myc. **A** Absorption titration of 8 μM biotinyl cNDI with addition of c-myc, **B** theoretical fit of UV/Vis titration data using the least squared method, and **C** ITC measurement of c-myc with addition of biotinyl cNDI in 50 mM KH_2_PO_4_-K_2_HPO_4_ buffer (pH 7.0)

Biotinyl cNDI (**1**) was synthesized by condensation of the amino group of cNDI (**2**) with the active ester of biotin as shown in Scheme 1. The absorption spectrum of **1** gives an absorption maximum at 383 nm and the addition of c-myc produced a hypochromic effect and red shift as shown in Fig. 2A. This result indicates that **1** is stacking bound to the G4 plane. A plot of the percentage change in absorbance at 383 nm versus c-myc concentration is shown in Fig. 2B gave binding constant *K* = 8.9 × 10^6^ M^−1^ with *n* = 2 with fitting by the Stootman’s theoretical Eq. (1) [13]. This result is consistent with that for cNDI derivatives previously reported [14]. This binding behavior was also evaluated by ITC measurements. The results are shown in Fig. 2C, and the binding parameters obtained by fitting are summarized in Table 2. The values obtained from the ITC measurements were *n* = 2 and *K* = 3.9 × 10^6^ M^−1^. The values obtained from absorbance changes were almost twice as high as those from ITC measurement, but the binding constants were all on the order of 10^6^ M^−1^. The G4 ligand available for the pulldown assay, which has already been reported [8–10], had a binding constant of the order of 10^6^ M^−1^, indicating that this ligand can also be used for the pulldown assay. The thermodynamic parameters obtained indicate that the G4 binding of this ligand is enthalpically driven and is consistent with previous examples [15]. In particular, the entropy was favorable in this system because the ligand has a cyclic structure, so the structural changes before and after binding are small, and the entropy term is thought to be due to the dehydration effect.

**Table 2:** Binding parameters of **1** with the several G4 DNAs based on ITC measurement

DNAs	10^−6^* K*/M^−1^	Δ*G*/kcal mol^−1^	Δ*H*/kcal mol^−1^	-*T*Δ*S*/kcal mol^−1^	n
c-myc	3.9	− 9.0	− 7.4	− 1.6	2
c-kit	0.20	− 7.2	− 7.6	0.40	1
VEGF	0.82	− 8.1	− 8.4	0.32	2
TA-core	2.1	− 8.6	− 7.0	− 1.7	2

The binding behavior of **1** to other G4 structures was carried out based on absorption titration (Fig. S3-4, Table S1) or ITC measurements (Fig. S5) and thermodynamic data based on ITC measurements are summarized in Table 2. TA-core showed a binding constant of about the same order of magnitude, although slightly lower than that of c-myc. The binding capacity for c-kit and VEGF was an order of magnitude lower. The reason for this is not clear but may be due to steric hindrance between the liker part of **1** and the G4 loop of c-kit or VEGF. This behavior is supported by the slightly unfavorable entropy term in the binding energy. The binding constants obtained from the absorption spectra were larger than those obtained from the ITC spectra for all DNAs, but the order of binding ability by DNA was the same. For double-stranded DNA, binding constants could not be obtained from ITC, but were estimated from small changes in the absorption spectrum to be less than 1/100. In any case, it is clear that **1** can function as a highly selective G4 ligand.

### Effect of G4 structure on binding of *1* and its stability ability

**Fig. 3:**
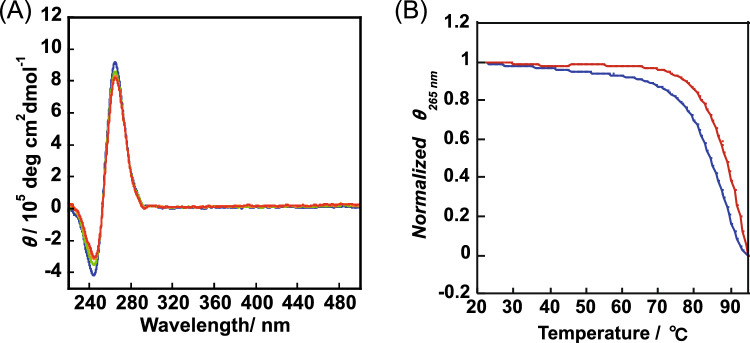
**A** CD spectra of c-myc upon addition of **1** (0, 1, 2, or 3 eq. from upper to bottom), and **B** Tm measurement of 1.5 mM c-myc in the absence or presence of **1** (1:2 molar ratio) in 50 mM KH_2_PO_4_-K_2_HPO_4_ buffer

Figure 3A shows the CD spectrum of c-myc, which shows a negative cotton effect at 240 nm and a positive cotton effect at 260 nm, indicating a parallel structure. CD spectra showed that c-kit and VGEF were also parallel structures, while TA-core was a hybrid structure (Fig. S6), which is consistent with previous papers [16]. The addition of **1** to c-myc did not cause significant CD spectral changes as in the case of other G4s (Fig. 3A). This indicates that **1** does not disrupt the G4 structure when bound to G4. The T_m_ curve was obtained by adding **1** to G4 DNA at a molar ratio of 1:2 and measuring the temperature dependence of CD intensity (Fig. 3B and Fig. S7) for the example of c-myc. The stabilizing effect of **1** on other G4 DNA was also observed and the results are summarized in Table 3. All of the G4 DNAs showed the stabilizing ability of **1**, whereas no stabilizing effect was observed for double-stranded DNA. This indicates that **1** is a superior G4 capture ligand.

**Table 3:** Thermal stability enhancement of **1** for G-quadruplex structure

DNA	Tm (0 eq.)/^o^C	Tm (2 eq.)/^°^C	ΔTm/^°^C
c-myc	≧86.7	≧ 93.3	≧5.4
VEGF	≧70.0	≧77.0	≧7.0
TA-core	62.4	67.2	4.8
dsoligo	50.0	50.1	0.1

### Pulldown assay using G4 DNAs

**Fig. 4:**
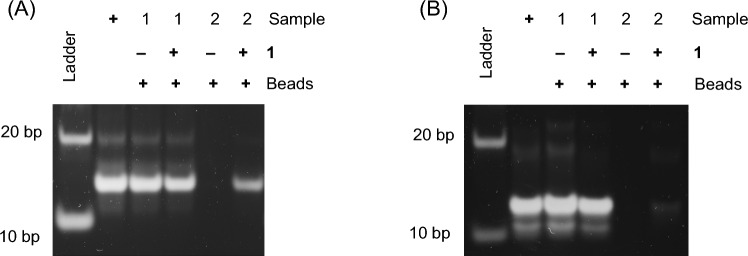
Gel electropherogram for sample 1 (supernatants after pull-down) and sample 2 (solutions after recovery) of 5 μM c-myc **A** and 5 μM dsoligo **B**

A pulldown assay was performed using c-myc DNA or dsoligo according to the procedure in Fig. 1. After treatment of the solution containing c-myc DNA with **1** and streptavidin magnetic beads (sample 1), the c-myc DNA captured on the magnetic beads was removed by collecting the beads with a magnet and subsequent treatment at 99 °C for 30 min (sample 2). The same procedure was performed without Ligand, **1**. Figure 4 shows the results of gel electrophoresis of the samples at each step. As shown in Fig. 4A, in the case of c-myc, a c-myc band cannot be observed in sample 2 without **1**. This indicates that c-myc is not collected by the beads without **1**. On the other hand, in the presence of **1**, c-myc could be collected by the beads treatment. A slight decrease in the c-myc band in sample 1 was confirmed, and the c-myc band was observed in sample 2. Although the recovery rate of the G4 pull down ligands reported for oligonucleotide targets has not been investigated much, L1Bio-7OTD, an oxazole telomestatin derivative [9], has been subjected to similar gel electrophoresis experiments. That recovery rate appeared to be comparable to that for **1**. Figure 4B shows the case of dsoligo, where dsoligo was not collected by the beads even in the presence of **1**. The results of the absorption spectrum measurement of the same sample are shown in Fig. S8, supporting the results of Fig. 4, but a component with a maximum absorption at 280 nm was observed because of the protein. This may be due to the stripping of streptavidin by the treatment of the beads. This result was confirmed by **1** can be used for the G4-specific pulldown assay shown in Fig. 1.

**Fig. 5:**
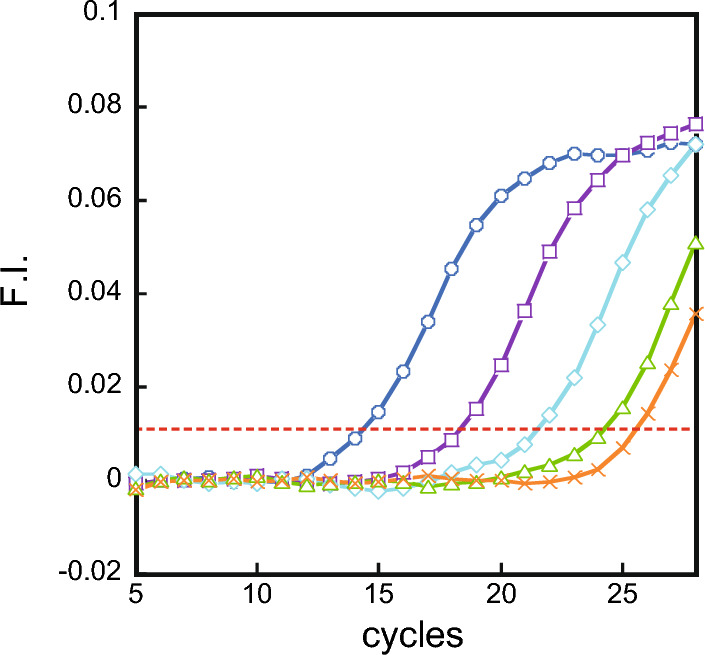
qPCR after pulldown of 120mer DNA fragment containing c-myc120 (2 fmol c-myc120; ○, 200 amol c-myc120; □, 20 amol c-myc120; ◇, 2 amol c-myc120; △) or c-myc-mut95 (2 fmol; ×) with threshold (0.0117)

The pulldown assay was performed using c-myc120, which contains single c-myc sequence in 120 mer, and c-myc-mut95, which does not. This experiment was performed using samples with concentrations of 2 fmol, 200 amol, 20 amol, or 2 amol for c-myc120. The experiment with c-myc-mut95 was performed at 2 fmol, the highest concentration in the c-myc120 experiment. The samples were distributed by Pulldown and subjected to qPCR (Fig. 5). A calibration curve for c-myc120 was generated by qPCR (Fig. S9 and Table S2) to estimate the recovery of the pulldown assay. As a result, more than 50% recovery was achieved. In particular, 95% recovery was achieved with 2 amol. In contrast, no recovery was achieved for those without G4 (the slight increase in fluorescence at higher cycle numbers is due to primer dimer generation in PCR).

These results indicate that G4 can be captured by **1** even from long DNA fragments with G4.

## Conclusions

Here, we succeeded in synthesizing a new biotinyl cNDI, **1**, which is a biotinylated version of cNDI with high G4 selectivity. **1** had a high binding and stabilizing ability to G4, and biotin could be added while maintaining the properties of cNDI. In addition, G4 was successfully extracted selectively by pulldown using **1** coupled with streptavidin magnetic beads. Here, we have not even confirmed the sequence selectivity of the pulldown ability of this ligand. Recently, the localization of G4 and the binding sites of G4-ligand have been detected genome-wide using NGS, suggesting the influence of small molecules on G4 formation under in vivo conditions [17, 18]. In the future, it will be important to investigate the effects of various ligands, including the ligand developed in this study, on G4 formation in the drug design of G4-binding drugs. Chem-map, a method for mapping small molecules that interact with DNA or chromatin-related proteins in cells by using biotinyl ligands and applying the CUT&Tag method, was reported [17]. **1** can also be used in Chem-map as a biotinylated G4 ligand. We believe that **1** can be expanded into Chem-map and provide valuable information on DNA interactions in cells that can contribute to drug development.

### Supplementary Information

Below is the link to the electronic supplementary material.

Supplementary file1 (PDF 516 kb)

## Data Availability

The data supporting the findings of this study are available within the paper and its Supplementary Information files. Should any raw data files be needed in another format they are available from the corresponding author upon reasonable request.
