# Supplementary material for: Biotinylated cyclic naphthalene diimide as a searching tool for G4 sites on the genome
Source: Anal Sci. 2024 Apr 12;40(5):943–50. doi: 10.1007/s44211-024-00551-5 (PMC11035424; doi:10.1007/s44211-024-00551-5)
Supplement: Supplementary file 1 — Supplementary file1 (PDF 516 kb) [file 44211_2024_551_MOESM1_ESM.pdf]

Supporting Information

Biotinylated cyclic naphthalene diimide as a searching tool for G4 sites on the genome

Satoshi FUJII,[*1†] Shinobu SATO,[*2†] Ryuki HIDAKA,[*2] and Shigeori TAKENAKA[*2]

[*1]*Department of Bioscience and Bioinformatics, Kyushu Institute of Technology, 680-4 Kawazu, Iizuka-shi, Fukuoka 820-8502, Japan*
[*2]*Department of Applied Chemistry, Kyushu Institute of Technology, 1-1 Sensui-cho, Tobata-ku, Kitakyushu-shi, Fukuoka 804-8550, Japan*

[†] To whom correspondence should be addressed.
E-mail: sfujii@bio.kyutech.ac.jp

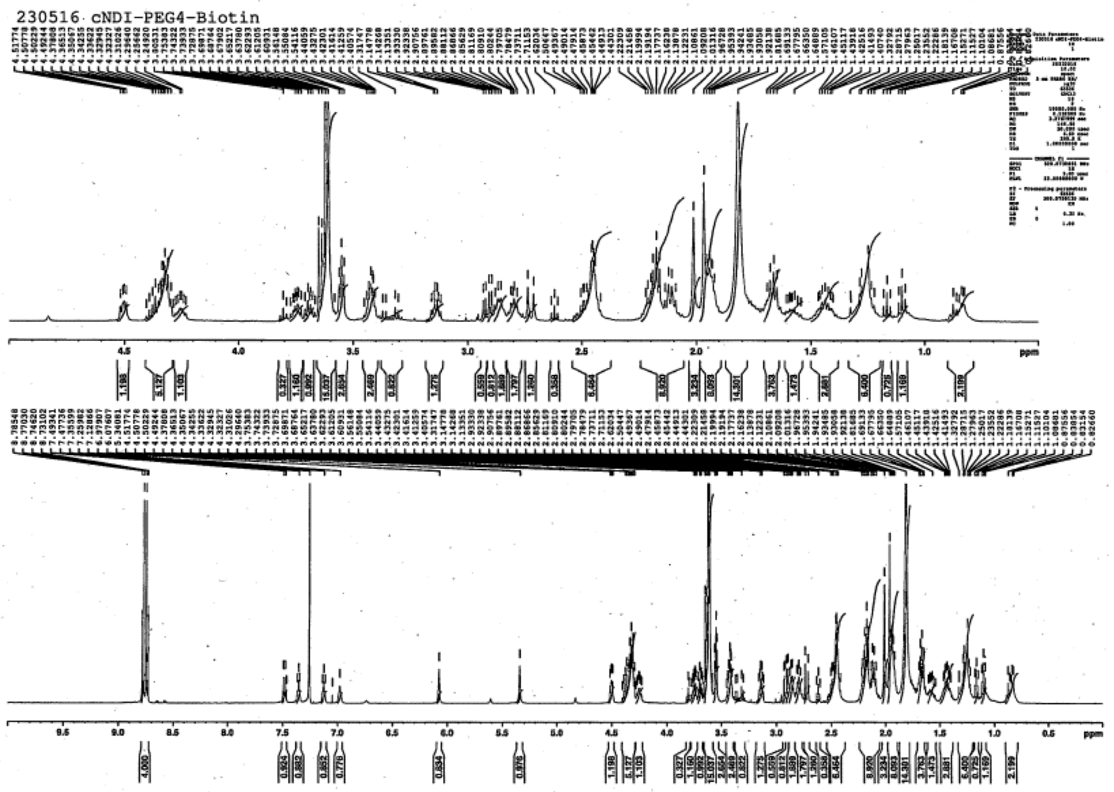

Fig. S1. ^{1}H-NMR spectra of **1** in $CDCl_3$ using TMS as an internal standard.

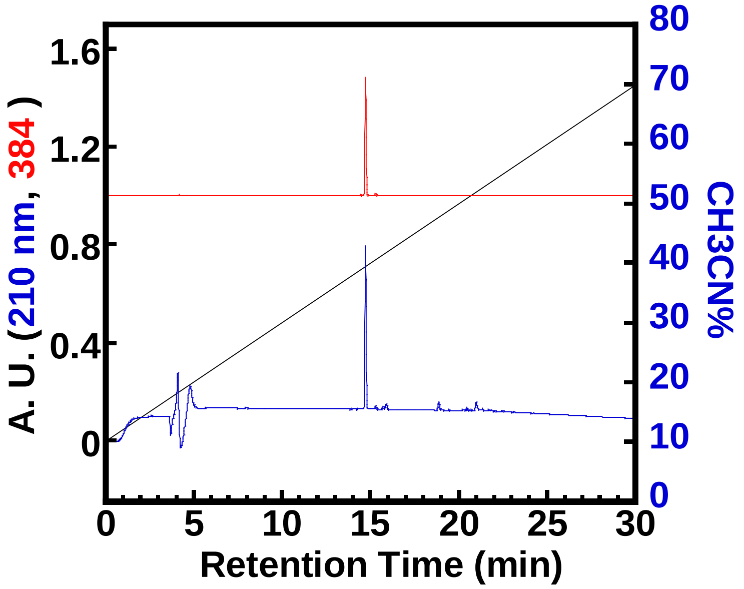

Fig. S2. Reversed phase HPLC of **1**. The concentration of acetonitrile (CH_3CN) was changed from 7% to 70% in water containing 0.1% CF_3COOH over 30 min at 40 ˚C.

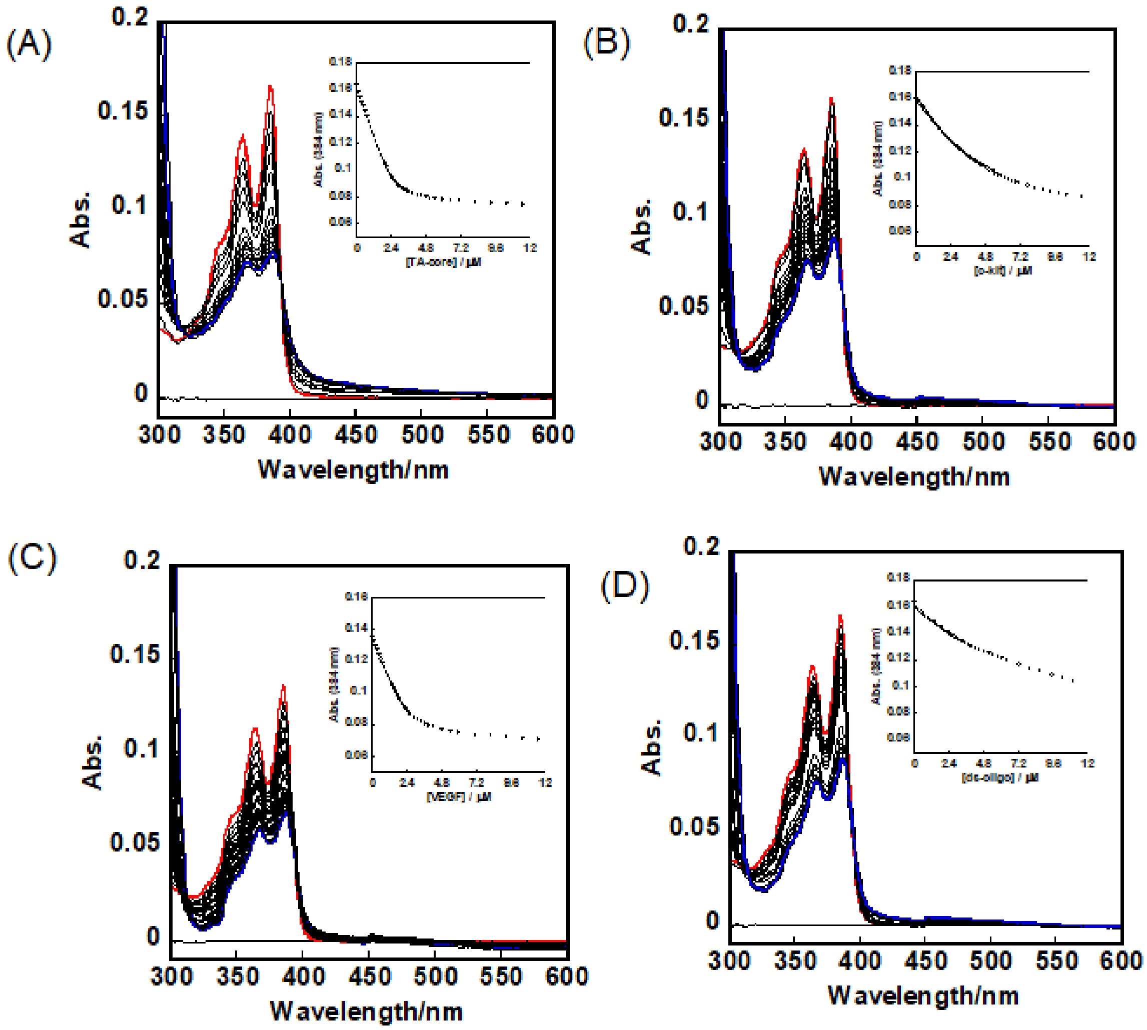

Fig. S3. UV spectra of 8 μM (A, B, or D) or 5 μM (C) of **1** in the presence of from 0 μM to 12 μM TA-core (A), c-kit (B), VGEF (C), or ds-oligo (D) in 50mM H_2KPO_4 HK_2PO_4 buffer (pH 7.0) at 25 °C. Absorption change upon addition of DNA was inserted in the graph.

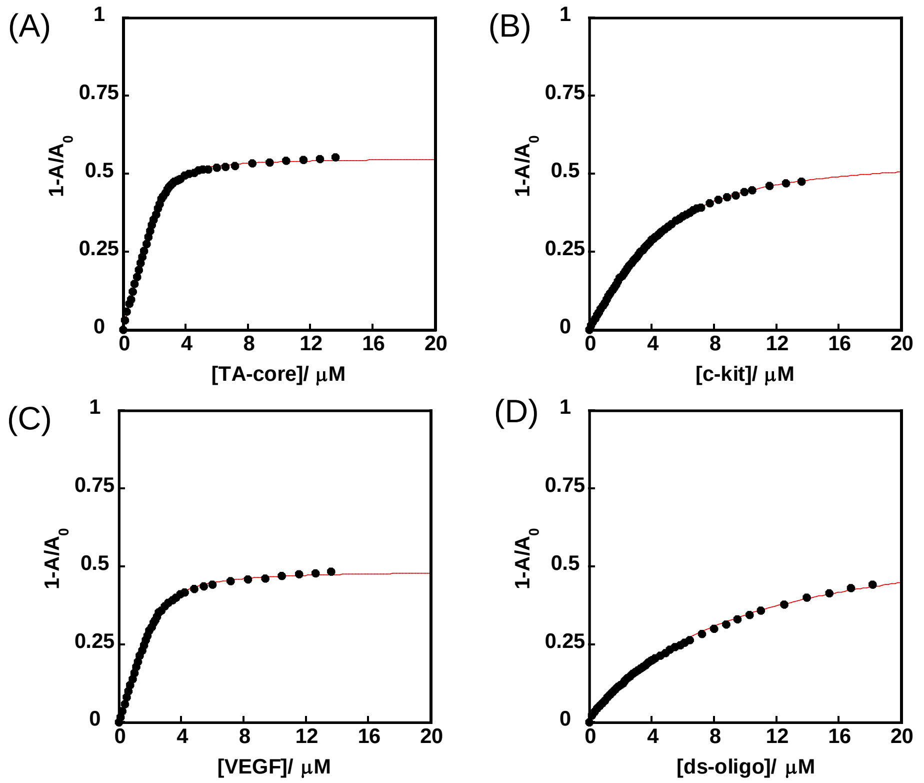

Fig. S4. Fitting plots of the interaction of **1** with TA-core (A), c-kit (B), VGEF (C), or ds-oligo (D) based on Stootman's equation (1) in 50mM H_2KPO_4 HK_2PO_4 buffer (pH 7.0) at 25 ^{o}C.

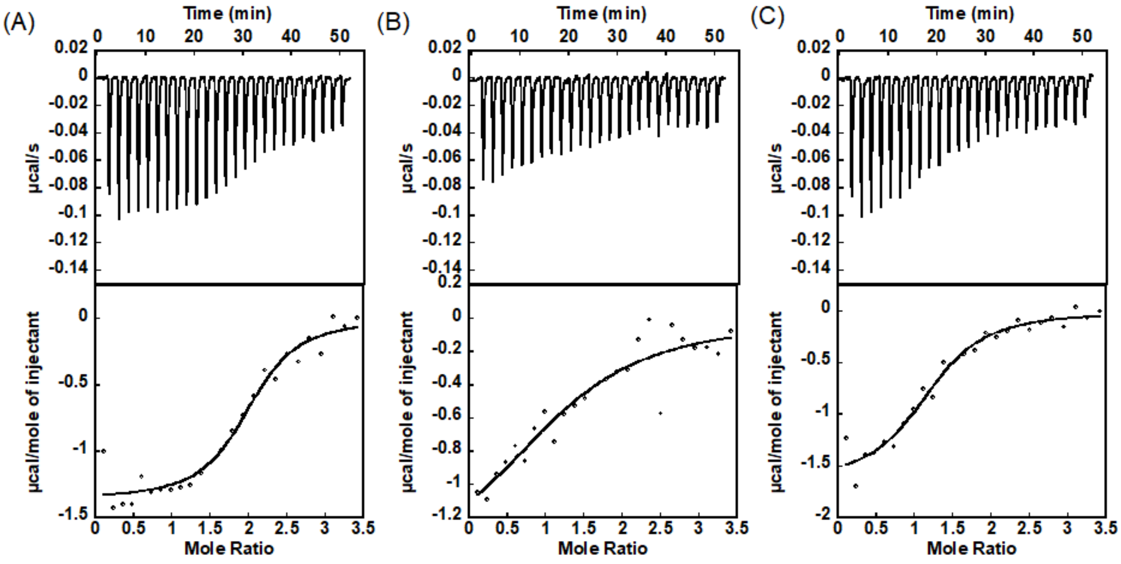

Fig. S5. ITC measurement of TA-core (A), c-kit (B), VGEF (C) with addition of **1** in 50 mM KH_2PO_4-K_2HPO_4 buffer.

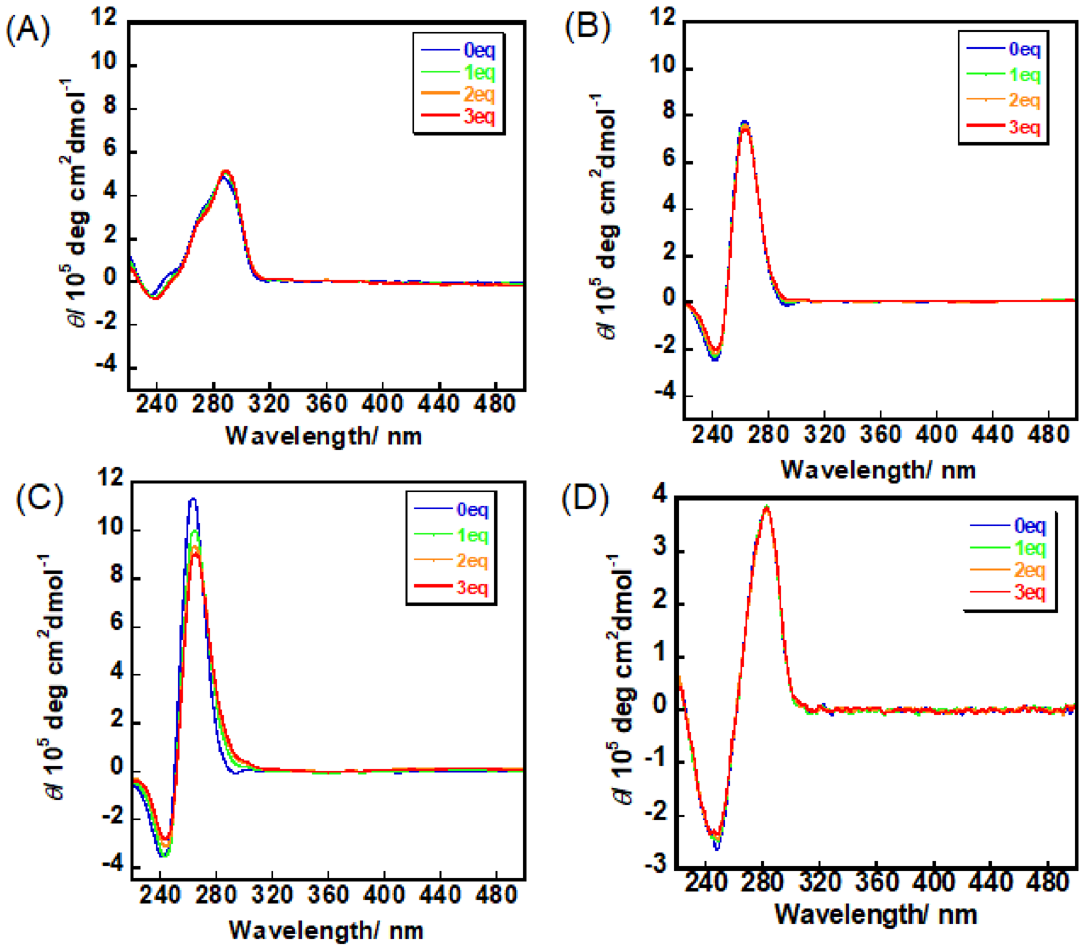

Fig. S6. Circular dichromic spectra of 1.5 μM TA-core (A), c-kit (B), VGEF (C), or ds-oligo (D) in 50mM H_2KPO_4 HK_2PO_4 buffer (pH 7.0) at 25 °C under 0, 1.5, 3.0 and 4.5 μM **1** at 25˚C.

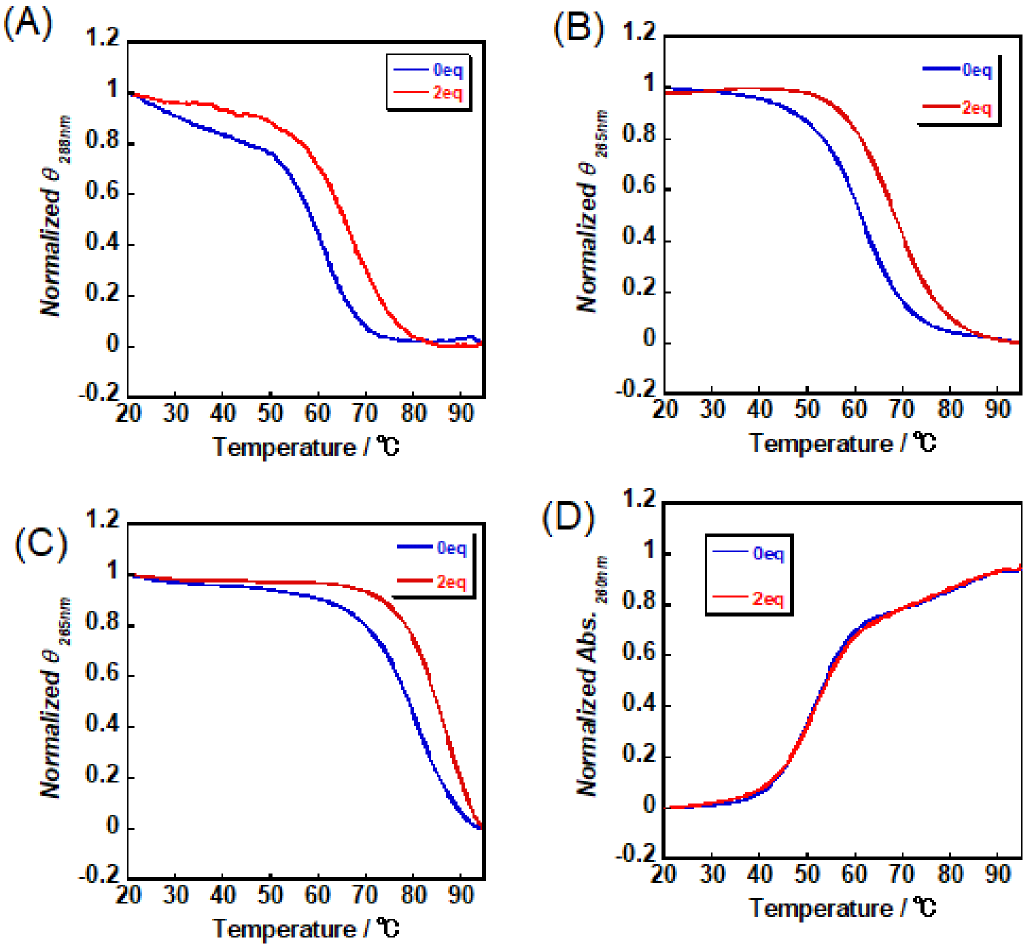

Fig. S7. Melting curves of 1.5 μM TA-core (A), c-kit (B), VGEF (C), or ds-oligo (D) with the absence (blue) or presence of 3.0 μM **1** in 50mM H_2KPO_4 HK_2PO_4 buffer (pH 7.0) at 25 °C

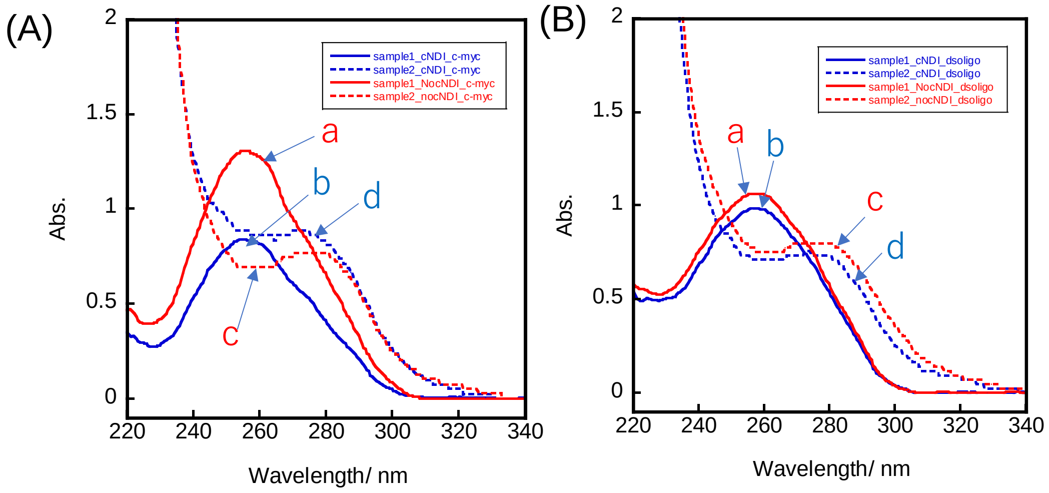

	a	b	c	d
Sample	1	1	2	2
cNDI	–	+	–	+
Beads	+	+	+	+

Fig. S8. Absorption spectra for sample 1 (supernatants after pull-down, solid line) and sample 2 (solutions after recovery, dotted line) of 5 μM c-myc (A) and 5 μM dsoligo (B) in the presence (blue) or absence (red) of cNDI-biotin.

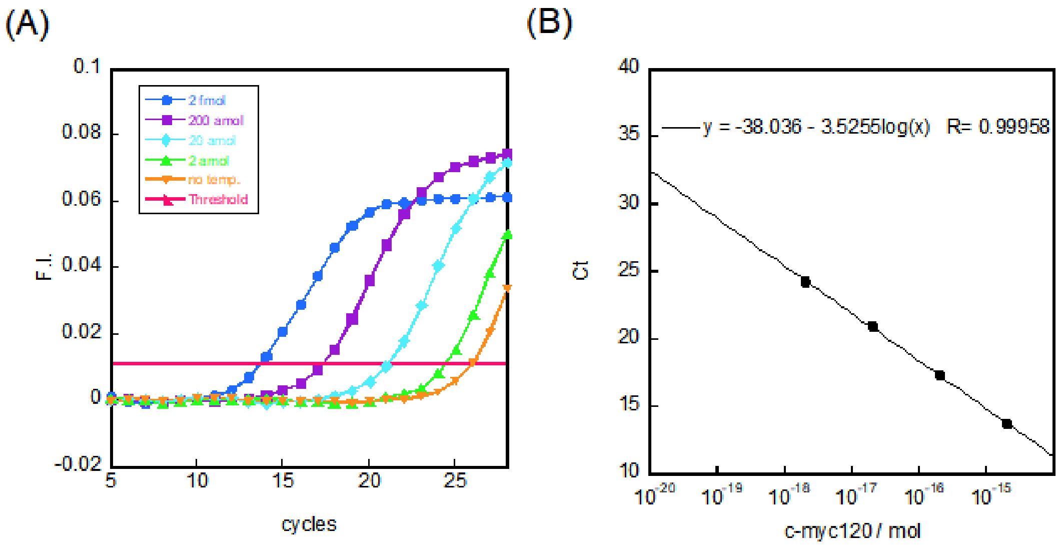

Fig. S9. (A) Result of qPCR for c-myc120 standard curve with the threshold 0.01117, (B) the standard curve for c-myc120.

Table S1 Binding parameters of **1** with the several G4 DNAs based on absorption titrations

DNAs	K_a /$10^6 M^{-1}$	n	Hypochromicity /%
c-myc	8.9	2	46
c-kit	0.42	1	48
VEGF	1.6	2	48
TA-core	2.5	2	55
ds-oligo	0.023	2	44

Table S2 Recovery rate of pull down assay for c-myc120

c-myc120*	2 fmol	200 amol	20 amol	2 amol
Ct	14.44	18.31	21.73	24.43
Recovered DNA	1.3 fmol	100 amol	11 amol	1.9 amol
Recovery rate/%	65	50	55	95

* The amount of c-myc120 indicates the amount when 100% recovery is achieved.
